# Dietary Determinants of Metabolic and Gut Microbial Health in Patients with Inflammatory Bowel Disease

**DOI:** 10.3390/nu16193233

**Published:** 2024-09-24

**Authors:** Gabrielle Wark, Nadeem O. Kaakoush, Dorit Samocha-Bonet, Simon Ghaly, Mark Danta

**Affiliations:** 1 School of Clinical Medicine, St Vincent’s Healthcare Campus, Faculty of Medicine & Health, University of New South Wales, Sydney 2010, Australia; gabrielle.wark@health.nsw.gov.au (G.W.); d.samochabonet@garvan.org.au (D.S.-B.); simon.ghaly@svha.org.au (S.G.); 2 Department of Gastroenterology and Hepatology, St Vincent’s Hospital, Sydney 2010, Australia; 3 School of Biomedical Sciences, Faculty of Medicine & Health, University of New South Wales, Sydney 2052, Australia; n.kaakoush@unsw.edu.au; 4 Garvan Institute of Medical Research, Sydney 2010, Australia

**Keywords:** inflammatory bowel diseases, Crohn’s disease, ulcerative colitis, metabolic risk, microbiota, diet, processed meat, dietary fibre, flare

## Abstract

*Background:* Diet has been linked to gut dysbiosis and the onset, course, and response to treatment of patients with IBD and metabolic disease. *Methods*: This single-centre prospective case-control study investigated the relationship between dietary intake, metabolic profile, and stool microbial composition in 57 individuals with IBD in clinical remission and 24 healthy individuals (HC). Participants’ baseline anthropometric measurements, serum metabolic parameters, lipid profiles, and oral and stool samples for microbiota testing were collected. Their dietary intake and physical activity were documented. A partially corrected correlation was performed to examine the associations between variables and *p*-values adjusted for multiple comparisons using the Benjamini–Hochberg equation (adj-*p*). *Results:* In participants with IBD, the intake of saturated fat correlated positively, and the intake of dietary fibre correlated negatively with anthropometric indices (saturated fat and BMI: r = 0.37, adj-*p* = 0.04, fibre and BMI: r = −0.45, adj-*p* = 0.01). Higher anthropometric indices were associated with poorer glucose control and a less favourable serum lipid profile (BMI and insulin: r = 0.48, *p* < 0.01, WHR and triglycerides: r = 0.57, *p* < 0.01). The stool microbiota of participants in the IBD group was less diverse and more similar to their oral microbiota than was observed in the HC group (Mann–Whitney U test *p* = 0.03). Within the IBD group, a higher intake of added sugar and processed meat and a higher serum insulin level was associated with lower stool microbial alpha diversity (processed meat intake and Shannon’s diversity: r = −0.43, adj-*p* = 0.02; added sugar and Shannon’s diversity: r = −0.39, adj-*p* = 0.03; insulin and Shannon’s diversity: r = −0.45, adj-*p* = 0.02). Neither the dietary intake nor stool microbial composition correlated with the risk of disease flaring. *Conclusions*: Our findings suggest that dietary intake is associated with the metabolic health and gut microbial composition of IBD patients.

## 1. Introduction

The prevalence of obesity among patients with inflammatory bowel disease (IBD) has increased two- to three-fold in recent decades, as is the case in the general population. In the US, obesity prevalence in patients with IBD rose from 12.5% in the early 1990’s to 20.2% in 2005–2010 [1]. This trend is set to continue.

The rise in obesity in patients with IBD is of particular importance, given obesity is associated with multiple serious health conditions, including type 2 diabetes (T2DM), ischemic vascular diseases, metabolic-associated steatotic liver disease (MASLD), hypertension, dyslipidemia, malignancies including colorectal cancer, joint disorders, and depression [2]. Patients with IBD are already at an increased risk of these conditions due to the concurrent burden of chronic inflammation, and the presence of obesity may further compound this risk [3,4].

Several common treatments used in the management of patients with IBD may also contribute to cardiometabolic risk. Corticosteroids are associated with weight gain and impaired glucose tolerance [5]. Janus kinase (JAK) inhibitors have been associated with an increased risk of major adverse cardiac events (MACE) when used in the treatment of other inflammatory conditions, such as rheumatoid arthritis and, more recently, Ulcerative Colitis (UC); this risk was higher in those with risk factors for cardiovascular disease [6,7]. The presence of obesity and its associated metabolic risk would make the use of JAK inhibitor medications in patients with IBD less safe [6].

Central adiposity and increased BMI may also impact the disease course and treatment of IBD. Individuals living with IBD and obesity were found to have lower biological medication trough levels, higher rates of treatment failure with these medications, and a greater need for surgery [8].

These people suffer an increased incidence of post-operative complications, require a longer hospital stay, incur higher hospital costs, are more likely to report extra intestinal manifestations (EIM) of IBD and are more likely to have a comorbid psychological diagnosis [9]. Maintaining a healthy body weight in patients with IBD may help to optimise IBD management. Although dietary predictors of an unhealthy weight in the general population are known, these predictors have not been specifically studied in IBD populations.

Obesity and IBD have several lifestyle and dietary risk factors in common. A sedentary lifestyle and a diet with a higher content of ultra-processed food, red meat, and added sugar has been associated with the development of IBD, while the intake of *n*-3 polyunsaturated fatty acids (PUFA) and fibre has been associated with a reduced risk [10,11,12]. Many of the dietary factors associated with the development of IBD have also been associated with the development of obesity; however, despite these shared dietary risk factors, there is a paucity of studies looking at the relationships between diet and metabolic health within IBD cohorts.

The gastrointestinal microbiota plays a crucial role in the interaction between the environment and host immune and metabolic responses. It may have a significant impact on the development and progression of both IBD and obesity. The gut microbiome in IBD is characterised by decreased bacterial microbial diversity, an increase in invasive, adherent species with the potential to cause disease in at-risk individuals (labelled pathobionts), and a reduction in favourable short-chain fatty acid (SCFA) producing species, which have been associated with a gut mucosal barrier function and colonocyte (epithelial cells that line the colon) health [13]. Emerging data also suggest that the expansion of oral-associated bacteria in the gut may be a feature of IBD [14]. Stool microbial dysbiosis (an imbalance in bacterial composition) correlates with IBD activity and the response to treatment [15,16,17].

The gut microbiota of people living with obesity and metabolic diseases such as type 2 diabetes have also been found to exhibit an altered microbial composition when compared to cohorts of people with healthy weight [18,19,20]. These changes resemble those observed in patients with IBD. The gut microbiome may also impact the management of these conditions. Research has been conducted on IBD and those with metabolic disease as separate entities; however, no prospective studies have examined the association between the diet, metabolic profile, and stool microbial diversity of patients with IBD.

This study aimed to prospectively characterise the dietary, lifestyle, and stool microbial parameters that are associated with metabolic risk factors in IBD patients.

## 2. Materials and Methods

### 2.1. Participants

This prospective case-control study recruited participants with IBD from the outpatient clinic at St Vincent’s Hospital, Sydney, Australia, a tertiary referral centre, and healthy (HC) participants were recruited via hospital/precinct-based poster advertising.

Inclusion criteria for the IBD group were adults (≥18 years) with a history of colonic distribution of Crohn’s disease (L2 or L3 as defined by the Montreal classification of CD) or Ulcerative Colitis extending beyond the rectum (≥E2 as defined by Montreal Classification of UC) [21]. Patients with isolated small bowel Crohn’s disease (L1) were excluded, given the distinct genetic, microbial, and phenotypic profile that may make this disease distribution a unique entity [22]. To avoid the confounding influence of active disease on dietary intake and microbial composition, participants were required to be in stable, corticosteroid-free clinical remission (defined by Partial Mayo Score (PMS) ≤ 2 with no sub score >1 or Crohn’s Disease Activity Index (CDAI) <150) for the preceding 3 months. Diagnosis and medication history were ascertained by participant interviews and a review of medical records, which included previous endoscopy reports. Exclusion criteria included antibiotic or steroid use in the preceding 3 months, a diagnosis of diabetes, ulcerative proctitis, or prior total colectomy.

The HC group included adults ≥18 years of age and excluded those with gastrointestinal symptoms, a diagnosis of diabetes/impaired glucose tolerance, the presence of a significant systemic disease, and women who were pregnant or breastfeeding.

This was a hypothesis-building pilot study. The study protocol and participant information documents were approved by the St. Vincent’s Hospital Human Research Ethics Committee (HREC/18/SVH/105). The study was conducted in accordance with the 2013 (Fortaleza) revision of the 1964 Declaration of Helsinki. Informed consent was obtained from all subjects involved in the study.

### 2.2. Clinical Data Collection and Processing

At the baseline, anthropometric indices recorded were weight, height, body mass index (BMI), waist circumference (WC), hip circumference (HC) and waist-to-hip ratio (WHR). BMI classifications used were as follows: underweight BMI < 18.5 kg/m^2^, healthy weight BMI 18.5–24.9 kg/m^2^, overweight 25–29.9 kg/m^2^, obese ≥ 30 kg/m^2^ and consideration to ethnicity-specific cutoffs was given. Baseline fasting blood samples were collected for measurement of serum glucose, insulin, glycated hemoglobin (HbA1c), lipid profile and c-reactive protein (CRP). Oral and stool samples were collected for the assessment of microbial composition, further detailed below; stool samples were additionally tested for fecal calprotectin (FC) levels, measured by standard enzyme-linked immunosorbent assay (ELISA) testing. Blood samples were processed using standard protocols for routine clinical care, and diets and physical activity were monitored for 10 days. Participants with IBD were followed up for evidence of clinical flare, defined by clinical symptoms (PMS > 2 and CDAI > 150), necessitating an escalation of treatment.

### 2.3. Dietary Information

Participants used a diet diary smartphone app (Easy Diet Diary, Xyris, Brisbane, Australia) to record their diet. The incomplete first and last days were omitted from the analysis, resulting in 8 days of diet diary data for review for each study participant. The nutritional breakdown was analysed using FoodWorks 10 software (Xyris, Australia). Diet quality was assessed using the modified Healthy Eating Index for Australian Adults (HEIFA-2013) [23], with higher scores reflecting a dietary intake more consistent with healthy eating guidelines. Water intake was not recorded; as such, this component was omitted from the score. Discretionary foods (energy-dense, nutrient-poor foods) were calculated in accordance with the Australian healthy eating guidelines [24].

### 2.4. Activity Monitoring, Calculation of Basal Metabolic Rate, and Assessment of Dietary Under-Reporting

Participants wore an activity monitor (activPAL4, PAL Technologies, Glasgow, UK). The basal metabolic rate (BMR) was calculated using the Schofield equation (based on age, gender, and body weight) [25]. Diet diary data were assessed for under-reporting using the Goldberg equation; the Physical Activity Level (PAL) = Energy Intake (EI)/BMR and is an assessment of the plausible energy intake required in a healthy population [26]. A PAL of 1.55 (CI 1.07–2.24) considered the minimum energy intake requirement for an individual with a sedentary job and some active leisure time, with dietary recordings below the lower-level confidence interval (CI) cutoff of <1.07 considered to be under-reporting.

### 2.5. Microbial Sampling and Analysis

Participants were provided with stool collection kits PSP Spin Stool DNA Plus Kit (Invitek Molecular, Berlin, Germany) and oral collection kits OMNIgene OMR-110 ORAL collection and stabilisation kit (DNA Genotek, OT, Canada) to self-collect stool and oral samples at the commencement of the monitoring period.

Stool bacterial DNA was extracted using the PSP Spin Stool DNA Plus Kit (Invitek Molecular, Berlin, Germany), and 16S rRNA gene amplicon sequencing was performed. The V4 region (515f/806r) of the rRNA genes (2 × 250 base pairs) was amplified using conserved primers and Illumina to generate libraries. The MiSeq platform (Illumina Inc, San Diego, CA, USA) was used. Sequencing was performed at the Ramaciotti Centre for Genomics (UNSW, Sydney, Australia). Microbial DNA was analysed using Mothur pipeline v1.44.2 (open-source software for bioinformatics processing) and vsearch v2.13.3 (open-source bioinformatics software). SILVA v132 was used for alignment, and RDP v18 was used for taxonomy. Low-sequence samples below the 5000 clean read threshold were removed from the analysis, and the remaining samples were subsampled to an equal number of 5937 reads.

Stool microbial alpha diversity measures (Margalef’s species richness, Pielou’s species evenness and Shannon’s diversity index) were calculated using Primer-e v6, and differences between the groups were tested using GraphPad Prism v9. Beta-diversity was assessed by comparing Bray–Curtis similarities across samples generated from the square-root, transformed from Operational Taxonomic Unit (OTU) relative abundances. Differences across the groups were visualised using a principal coordinate analysis (PCoA) and tested with an Analysis of Similarities (ANOSIM), permutational multivariate analysis ANOVA (PERMANOVA), and permutational analysis of dispersions (PERMDISP). The PERMANOVA model included the variables of age, BMI, insulin, HEIFA diet score, study arm, and sex.

### 2.6. Statistical Analyses

Continuous data that were normally distributed were expressed as the mean and standard deviation (SD), and between-group *t*-tests or a 2-way ANOVA was performed to test for differences between groups when comparing 1 or >1 variables, respectively. Data that did not follow a normal distribution were reported in the median and interquartile range (IQR), and the Mann–Whitney U or Kruskal–Wallis tests were used to compare for 1 or >1 variables, respectively. The Chi-squared test was used to examine differences between categorical variables. A partially corrected correlation, which controlled for the effects of the covariates of age, gender, and average daily energy intake, was used to examine for associations between meal features, metabolic parameters, and stool diversity metrics (IBM SPSS Version 27). Where multiple correlations with a single variable were made, an adjusted *p*-value was calculated to minimise the false discovery rate (FDR) by using the Benjamini–Hochberg procedure. An adjusted *p*-value (adj-*p*) < 0.05 was considered significant.

Bray–Curtis similarities between the oral and stool microbiotas of each individual were calculated using Primer-e v6 bioinformatics software. Differences in the similarities between healthy individuals and participants with IBD were tested using a Mann–Whitney U test calculated on GraphPad Prism v9 software.

## 3. Results

### 3.1. Participant Characteristics, Dietary Intake, and Disease Activity

**Figure 1:**
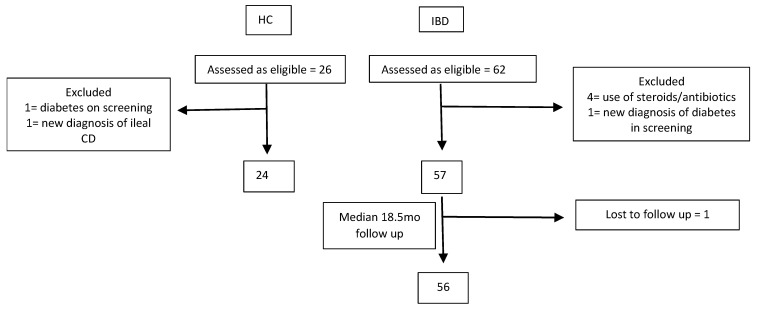
Flowchart of participants, with reasons for exclusion or withdrawal from the study. Legend: CD: Crohn’s disease, HC: healthy control, IBD: inflammatory bowel disease, mo: months.

Between March 2019 and April 2021, a total of 57 IBD (mean age 37.7 ± 11.4 years; 39% females; UC *n* = 26, CD *n* = 31) and 24 HC (mean age 38.0 ± 11.5; 46% females) participants were included in the study (Figure 1 and Table 1). There were no statistically significant differences between the IBD and HC cohorts in age, sex, anthropometric indices, metabolic and inflammatory indices. There were more smokers and ex-smokers in the IBD group (28% v 4%, *p* = 0.04).

There were 13/57 (22.8%) IBD participants who underreported their dietary intake and 2/57 (3.5%) who did not record dietary intake at all. In the HC group, all participants attempted to record dietary intake; however, 4/24 (16.7%) participants were underreported. The rates of under-reporting were not significantly different between groups (*p* = 0.35).

The IBD cohort averaged 18% fewer steps per day (9135 v 10,942 steps/day, *p* = 0.047, 95% CI 25 to 3588) and consumed an average of one less wholegrain serve per day (1.0 v 2.1, *p* = 0.01, 95% CI −1.7 to −0.5) (Table 2) than the HC group. The intake of other dietary components was comparable between groups.

The median IBD duration prior to study enrolment was 108 months (Interquartile range (IQR) 150 months) and the median FC was 17.4 µg/g (IQR 8–74 µg/g); a total of 45 (79.0%) participants were receiving biologic therapy, and 21 (36.8%) were on combination therapy with concurrent biologic medication and an immunomodulator (Thiopurine or Methotrexate) (Supplementary Table S1).

One IBD participant was lost to follow-up; the remaining 56 participants were followed up for a median of 18.5 months (IQR 12–24). Some participants flared up during this period 13/56 (23.2%), 7/56 (12.5%) within the first 12 months. Participants who flared up recorded a significantly higher baseline FC than those who did not (median FC 110 v 13 µg/g, *p* < 0.01). There was a lab processing error, which resulted in missing data for FC for 14 of the HC and 13 of the IBD patients. No dietary factor, anthropometric measure, or activity level (quantified by steps per day) correlated with the baseline FC level, the time to disease flare-up or the risk of a disease flare-up within the follow-up timeframe (Table 3).

**Figure 2:**
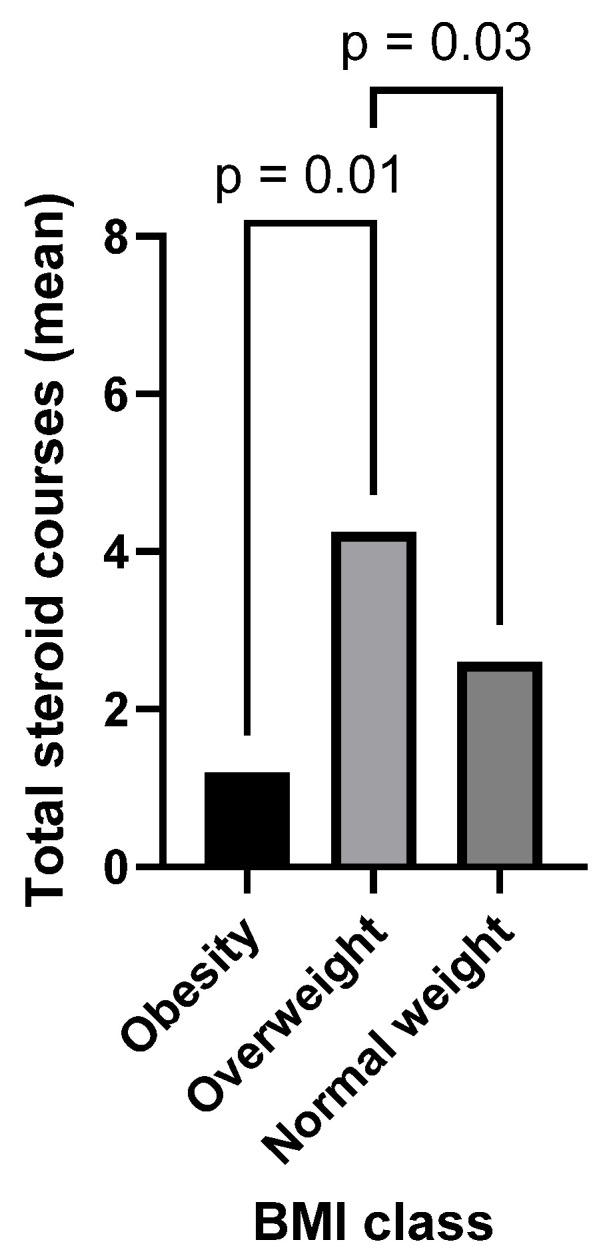
IBD group classified by BMI class. Legend: Bar graph displaying mean number of steroid courses per group. BMI: Body Mass Index class of participants in the IBD group: Obese (BMI ≥ 30, Overweight BMI 25–29.9, Normal weight BMI 18.5–24.9), *p*: *p*-value assessed using the Kruskal–Wallis test.

When IBD participants were classified by their BMIs into a healthy weight, overweight, and obese groups, there was a significant difference between the mean number of previous steroid courses administered to each group according to BMI classification. The overweight group had the highest previous steroid exposure, followed by the group in the healthy weight range (4.3 ± 3.0 courses versus 2.6 ± 2.5 courses, *p* 0.03) (Figure 2). The obese group had a lower average number of steroid courses than either the healthy weight or overweight participant groups (1.2 courses) (obese v overweight: *p* = 0.01, obese v healthy weight: *p* = 0.4). There were no significant correlations between participants’ number of previous steroid courses and their other anthropometric or metabolic parameters.

### 3.2. Associations between Diet, Anthropometric, and Metabolic Parameters

In the IBD cohort, after adjusting for age, gender, and average daily energy intake, the consumption of saturated fat was positively associated with the BMI and WC (saturated fat and BMI: r = 0.37, adj-*p* = 0.04, saturated fat and WC: r = 0.39, *adj-p =* 0.04), whilst a higher intake of fibre had a negative correlation with the BMI, WC, and WHR (fibre and BMI: r = −0.45, adj- *p* = 0.01, fibre and WC: r = −0.44, adj-*p* = 0.02, fibre and WHR: r = –0.45, adj-*p* = 0.02) (Table 4). In the HC group, there were significant positive associations between the intake of processed meat with the BMI and WC (processed meat and BMI: r = 0.59, adj-*p* = 0.05, processed meat and WC: r = 0.58, adj-*p* < 0.01).

In the IBD cohort, the average daily fibre intake was negatively associated with fasting glucose (r = −0.39, adj-*p =* 0.05) (Table 4). Otherwise, no other associations between the intake of specific dietary components and measures of glucose control reached statistical significance. There were no significant associations between the dietary intake and measures of glucose control in the HC group, nor were there significant associations between the dietary intake and serum lipid profile in the IBD or HC groups.

After adjusting for age and gender, we examined for correlations between participants’ anthropometric indices, their parameters quantifying glucose control, and serum lipid profiles (Table 5). In the IBD group, the BMI demonstrated a positive correlation with fasting insulin (r = 0.48, adj-*p* < 0.01) and a negative correlation with HDL (r = −0.44, adj-*p* < 0.01). WC and WHR were found to exhibit positive correlations with insulin (WC and insulin: r = 0.55, adj-*p* = <0.01, WHR and insulin r = 0.55, adj-*p* = <0.01) and triglycerides (WC and triglycerides: r = 0.36, adj-*p* = 0.04, WHR and triglycerides: r = 0.57, adj-*p* < 0.01).

In the HC group, WC had a positive correlation with LDL (r = 0.58, adj-*p* = 0.03) and cholesterol (r = 0.58, adj-*p* = 0.03).

We tested the associations between participants’ metabolic and anthropometric parameters and their blood and stool inflammatory markers. In the IBD cohort, there was a significant positive correlation between serum insulin, triglycerides, BMI, and WC with CRP and a negative correlation between HDL and CRP (Table 6).

There were no significant correlations between participants’ CRPs, anthropometric parameters, markers of glucose control or serum lipid profiles with CRPs in the HC group. There were no statistically significant correlations between anthropometric parameters, serum metabolic profiles, or serum CRPs with participants’ stool FC levels in either group.

There were no significant associations between the daily step count and anthropometric parameters nor fasting metabolic profiles in the IBD or HC groups.

### 3.3. Comparing Stool Microbiota Diversity between the IBD and HC Groups

The stool microbial alpha diversity of the IBD group was significantly lower than that of the HC cohort, with a lower species richness (IBD 21.4 v HC 26.8, *p* < 0.01, CI 2 to 9) and Shannon’s diversity index (IBD 3.1 v HC 3.4, *p =* 0.03, CI 0.03 to 0.6) (Supplementary Figure S1A,C). Species evenness was lower in the IBD group; however, this difference did not reach statistical significance (IBD 0.56 v HC 0.62, *p =* 0.16, CI −0.1 to 0.07) (Supplementary Figure S1B).

PERMANOVA analysis demonstrated a significant difference in the stool microbial composition between the IBD and HC groups, as measured by beta diversity (Bray–Curtis, Pseudo-F = 3.23, *df* = 1, 65, *p* < 0.01) (Supplementary Table S2A) and (Supplementary Figure S1D). There were no statistically significant associations between a participant’s stool microbial diversity metric and stool FC levels in either group (Table 3). In the IBD group, there was no association between the stool microbial diversity and the risk of a clinical disease flare-up at follow-up (Table 3).

**Figure 3:**
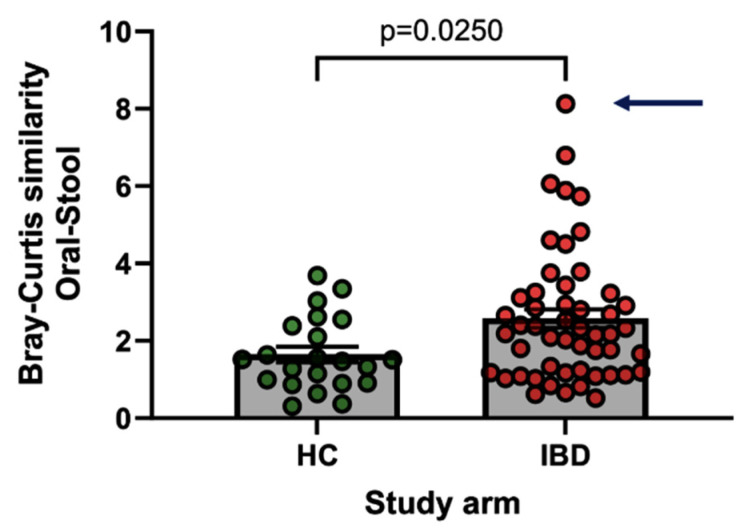
In the HC and IBD groups bar graphs which compare participants’ oral-stool microbiome bacterial similarities as measured by the Bray–Curtis similarities statistic, with a higher number representative of a more similar oral-stool microbial composition. Legend: Comparison was performed using Mann–Whitney U test following Shapiro-Wilk testing for data distribution. Arrow to indicate IBD participant who followed a ketogenic diet which included a high intake of processed meat.

Participants with IBD showed higher similarity between the oral and stool microbiotas compared to the HC group (Mann–Whitney U test, *p* = 0.025) (Figure 3). The magnitude of the similarity between the oral and stool microbiotas in participants in the IBD group was not significantly associated with dietary intake, anthropometric parameters, or the risk of subsequent disease flare-ups. Notably, one participant who followed a high-fat, low-carbohydrate diet and had a particularly high intake of processed meat had the most similar oral and stool microbiome of all participants (outlier participant: 1.7 serves/day v median of IBD cohort 0.1 serves/day, IQR 0–0.2 serves/day). This participant did not flare up during the follow-up period.

### 3.4. Associations between Stool Microbiota and Anthropometric, Dietary, Metabolic and Inflammatory Parameters

After controlling for age, gender and average dietary energy intake, in the IBD cohort, there was a negative correlation between the intake of processed meat and stool evenness and Shannon’s diversity index (processed meat and species evenness: r = −0.39, adj-*p* = 0.04; processed meat and Shannon’s diversity index: r = −0.43, *p* = 0.02) and a negative association between added sugar intake and Shannon’s diversity index (r = −0.39, adj-*p* = 0.03) (Table 7).

There was a negative correlation between serum insulin and participants’ stool microbiomial alpha diversity parameters of evenness and Shannon’s diversity index (insulin and species evenness: r = −0.45, adj-*p* = 0.02, insulin and Shannon’s diversity index: r = −0.45, *p* = 0.02). Higher BMI correlated with a lower Shannon’s diversity index (r = −0.36, adj-*p* = 0.04).

Further, serum CRP had a negative correlation with participants’ stool microbial evenness and Shannon’s diversity index (CRP and species evenness: r = −0.43, adj-*p* = 0.02, CRP and Shannon’s diversity index: r = 0.41, adj-*p* = 0.03).

Finding that BMIs and serum insulin and serum CRP concentrations were associated (Table 5), we performed a further analysis to additionally control for these factors. The correlations between insulin with evenness and Shannon’s diversity (insulin v evenness: r = −0.36, adj-*p* = 0.01, insulin v Shannon’s diversity: r = −0.31, adj-*p* = 0.03) and CRP and evenness (crp v evenness: r = −0.32, adj-*p* = 0.03) were maintained, whilst the association between CRP and Shannon’s diversity index (r = −0.28, adj-*p* = 0.05) BMI and Shannon’s diversity index was no longer significant (r = –0.05, adj-*p* = 0.74).

A PERMANOVA analysis was conducted to control the variables of the participant’s study group, age, BMI, insulin, sex, and average daily processed meat intake, and it was examined for factors associated with participant stool microbial beta diversity. When the IBD and HC cohorts were combined as one group (whole cohort), serum insulin levels had a statistically significant association with the stool microbial beta diversity (Pseud-F 1.66, *p* = 0.01, *df* 1, 65) (Supplementary Table S2A). The intake of processed meat exhibited a non-statistically significant trend to be associated with stool microbial beta diversity (Psedo-F 1.42, *p* = 0.06, *df* 1, 65).

When the IBD group was examined separately, there was a trend for both insulin and processed meat intake to be associated with the stool microbial beta diversity; however, this did not reach statistical significance (insulin: Pseudo-F 1.42, *p =* 0.07, *df* 1, 43, processed meat: Pseudo-F 1.38, *p =* 0.08, *df* 1, 43) (Supplementary Table S2B).

## 4. Discussion

In our study, a diet higher in saturated fat and lower in fibre in participants with IBD was associated with unfavourable metabolic health parameters, including BMIs. We also identified correlations between poorer anthropometric measures and adverse metabolic health, including suboptimal glucose control and elevated serum lipid profiles. These associations are of significance given the greater likelihood of overweight and obese people living with IBD experiencing biological medication treatment failure, requiring surgery, and experiencing more post-operative complications than those with a healthy weight. This is in addition to the fact that IBD patients are at a higher risk of cardiovascular disease, for which obesity, diabetes, and an unfavourable serum lipid profile are well-established risk factors [8]. Lastly, we report an association between dietary intake and markers of metabolic health and reduced stool microbial diversity.

We found that a higher serum insulin concentration was associated with a less diverse stool microbiota. A reduced stool microbial alpha diversity is a feature of IBD, a finding reproduced in our study, and is associated with the severity of colonic histologic inflammation, responses to treatments such as exclusive enteral nutrition (EEN), biologic medications, and fecal microbiota transplantation (FMT), as well as the risk of post-operative recurrence following ileocecal resection [15,16,17,27]. In non-IBD populations, a decreased stool alpha diversity has also been associated with obesity, impaired glucose tolerance, and the diminished effectiveness of the medical and surgical management of obesity, including bariatric surgery [18,19]. Conversely, a more diverse gut microbiota has been associated with improved metabolic outcomes.

In our study, in the IBD participant group, a higher intake of added sugar and processed meat correlated with a less diverse stool bacterial microbiota (processed meat v Shannon’s diversity index r = −0.43, *p* = 0.02). A recent study conducted in an American high school adolescent population reported that a higher intake of processed meat was associated with a lower stool alpha diversity, a lower abundance of the favourable SCFA-producing bacteria *Roseburia*, and a higher abundance of the pathobiont phylum, *Pseudomonadota* [28]. Animal models have demonstrated that a diet high in added sugar is linked with a decrease in gut microbial diversity and increased susceptibility to colitis, whilst human studies have linked added sugar consumption to the onset and activity of IBD [10,29,30].

The association between CRP and reduced stool evenness may be explained by the fact that systemic inflammation, be it related to IBD or metabolic disease, is characterised by reduced gut microbial diversity [31].

To our knowledge, the results of our study are the first time these metabolic and dietary correlations with stool microbial diversity have been reported in the setting of IBD.

Together, these associations suggest that optimising diet and metabolic health would avoid factors associated with reduced gut microbial diversity in patients with IBD and may be of benefit in the management of their gastrointestinal disease as well as in the prevention and management of metabolic diseases.

We found that the oral and stool microbiotas were more similar in participants in the IBD group than in the healthy cohort, suggesting a degree of ‘oralisation’ of the stool microbiota in IBD, which is consistent with the findings of other studies [14]. The fact that an IBD participant who followed a high-fat, low-carbohydrate diet, which included a high intake of processed meat (>8-fold the mean intake of IBD patients), had the most similar oral and stool microbiota of all the study participants suggests that a closer examination of dietary factors that may contribute to the oralisation of the gut microbiota is warranted.

Ultra-processed foods are characterised by highly saturated fat and low fibre content. These are industrial foods produced and designed to increase the product palatability and shelf life [32]. Ultra-processed foods have well-established associations with weight gain, obesity, and metabolic risk in the general population, including adverse cardiovascular outcomes, and have also been associated with the onset of IBD [10,32].

We observed that participants with IBD who consumed a diet characterised by a higher saturated fat and lower fibre content also had higher BMIs, WCs, and WHRs compared to those who consumed a more balanced diet. In the HC group, a greater intake of processed meat correlated with an increased WC and WHR.

Standardised, evidence-based dietary recommendations for IBD patients are lacking, and self-directed elimination diets and unnecessary dietary restriction is common in people with IBD, particularly the adoption of a lower fibre diet in the belief that this may improve gastrointestinal symptoms [33,34]. A higher intake of wholegrain foods and dietary fibre has been associated with a reduced risk of type 2 diabetes and cardiovascular disease and reduced risk of a Crohn’s flare-up at follow-up; thus, unnecessary dietary restrictions are a concern [35,36].

In our study, participants with IBD had a significantly lower intake of wholegrain foods compared with participants in the healthy cohort, with an average of one less serving per day (*p* < 0.01). This finding is consistent with a recent meta-analysis that found fibre and cereal consumption in people with IBD to be well below the national nutrient reference value recommendations [37].

In the IBD group, a higher average fibre intake was associated with a lower fasting glucose level. The findings of our study suggest that dietary intake is associated with the metabolic risk profile in patients with IBD, mirroring the established correlation in the general population; as such, unless a patient with IBD has significant stricturing small bowel disease, the restriction of dietary fibre should be avoided [21].

In the IBD group, a higher BMI, WC, and WHR were associated with poorer measures of glucose control and a less favourable serum lipid profile, including higher serum triglycerides and lower HDL cholesterol, associations that are well established in the general population [38]. Our findings support the notion that adhering closely to healthy eating guidelines, including avoiding the consumption of excess saturated fats and incorporating more fibre-rich wholegrain foods into the daily diet, may help achieve better gastrointestinal and metabolic health outcomes in people with IBD.

IBD may increase the risk of metabolic disease independently of traditional metabolic risk factors, as supported by a recent study, which reported that the rate of MASLD was higher in IBD compared with HC participants, independent of other metabolic risk factors, including BMI [39]. Serum CRP may increase in inflammatory conditions, such as IBD, with the level of elevation variable between individuals. This increase is influenced by age, disease location, the IBD subtype, and disease activity [40]. In the general population, an elevated CRP has been associated with the presence of metabolic syndrome and subsequent metabolic risk [41]. In our study, participants in the IBD cohort were in clinical remission with a median CRP and FC within the normal range; however, a higher CRP correlated with higher serum insulin and triglyceride levels, a higher BMI and waist circumference, and a lower HDL level, suggesting that in the absence of active gastrointestinal disease, elevated CRP may be a marker of metabolic risk.

BMI in the overweight range was associated with a higher cumulative number of steroid courses in our IBD cohort, a finding reported in previous studies [5]. A steroid-sparing treatment approach and proactive disease management with 5-Aminosalicytic Acids (5-ASAs), immunomodulators, and biological agents may be beneficial in controlling active disease, maintaining remission and to minimise the risk of weight gain associated with corticosteroid use [21]. The small number of IBD patients with obesity in our study (*n* = 5) had less steroid exposure than other IBD patients, a finding which may be explained by clinicians avoiding steroid use in patients living with obesity due to their known association with significant weight gain.

While both groups averaged a daily step count in or above the healthy target range (8000–10,000 steps/day), the IBD cohort averaged 18% fewer steps per day than the HC group (9135 versus 10,942 steps/day) [42]. Participants’ average daily step count was not associated with metabolic risk factors or the risk of an IBD flare-up within the follow-up period. This is consistent with previous studies, which have reported that physical activity is associated with a decreased risk of the development of IBD but not with subsequent flare-up risk [43]. Although exercise has not been shown to improve disease activity in established IBD, it may be beneficial to improve quality of life, reduce fatigue, and improve bone mineral density [44]. Prescribed exercise programs have been shown to assist in weight loss in non-IBD cohorts living with obesity [45]. Encouraging IBD patients, particularly those who are overweight or obese, to engage in regular exercise may be beneficial in the management of their weight and subsequent cardiometabolic risk.

No dietary factor, measure of metabolic health, or stool microbial diversity parameter predicted the risk of future IBD flare-ups in our cohort. To date, larger observational studies have reported that in UC, the avoidance of red and processed meat and a higher ratio of dietary *n*-3 PUFA to *n*-6 PUFA is associated with remission, whereas in CD, higher consumption of dietary fibre is associated with reduced risk of flare-ups [46]. Studies reporting microbial associations with future flare-up risk are small; however, research in the area is ongoing [27,47,48].

## 5. Limitations and Future Directions

The findings of this single-centre cohort study may not be generalisable to patients from other geographical regions with different local dietary practices. We reported on a relatively small sample size, particularly in the HC group, with missing data due to COVID-19 interruptions impacting the collection of some parameters, which reduced our ability to detect significant associations that have been established in larger groups. Dietary under-reporting was present; however, the rate of under-reporting was similar across groups and is an issue common to all self-reported dietary data. The study was observational rather than interventional, which limits the ability to establish causal relationships but is nonetheless hypothesis-building.

Our study analysed the microbiome using 16s rRNA amplicon sequencing, which provided a meaningful metric of overall microbial diversity to address our study aim. In future studies, metagenomic sequencing and metabolomic analysis to provide further detail in characterising microbial composition and downstream biological functions would be of interest [49].

Our study’s median follow-up period to detect clinical flare-ups was 18.5 months; however, longer-term follow-ups would be necessary to detect the emergence of the chronically natural complications of metabolic disease.

With these limitations in mind, it would be advantageous to perform a larger, multicenter study that included sites across different geographic locations, therefore encompassing different local diets, and with longer follow-ups to better capture metabolic outcomes, it would be useful to further explore the links between dietary intake, gut microbiome, and metabolic disease within IBD cohorts.

## 6. Conclusions

Our study highlighted the complex interrelationships between diets, anthropometric measures, glucose control, systemic inflammation, and the gut microbiota in patients with IBD. It emphasised the importance of avoiding unnecessary restrictions on dietary fibre, given the reported correlations with markers of metabolic health. Our findings support the hypothesis that diets may modulate both metabolic risk factors and gut microbial composition in IBD patients. Larger studies with longer follow-up periods are warranted to better inform dietary advice to IBD patients, with the aim of benefiting the management of their gastrointestinal illness as well as optimising their overall metabolic health.

## Figures and Tables

**Table 1:** HC and IBD group participant demographic, anthropometric and metabolic characteristics.

Parameter	HC	CI (95%)	IBD	CI (95%)	*p*
Number	24		57		
Age (mean, years)	38.0	33.1–42.8	38.7	35.7–41.7	0.79
Female sex (*n*)	11		22		0.72
Smoking Status					
Current	1 (4%)		5 (9%)		0.04 *
Past	0 (0%)		11 (19%)		
Never	23 (96%)		41 (72%)		
Anthropometric measures					
BMI (kg/m^2^)	24.8 ± 2.9		25.2 ± 4.3		0.63
BMI 18.5–25	12/25 (48%)		27/57 (47.4%)		
BMI > 25	12/25 (48%)		25/57 (44%)		
BMI > 30	1/25 (4%)		5/57 (9%)		
Waist circumference (cm)	81	77–85	84	79–88	0.42
Waist-to-Hip Ratio	0.9	0.8–0.9	0.9	0.8–0.9	0.65
Laboratory/Biochemical tests					
Hemoglobin (g/L)	139.5	134.3–144.6	141.4	139.0–143.9	0.44
Fasting plasma glucose (mmol/L)	4.4	4.2–4.6	4.5	4.3–4.7	0.4
HbA1c (%)	5.2	5.0–5.3	4.9	4.8–5.0	<0.01 *
Insulin (mU/L)	4.8	3.9–5.8	6.2	5.1–7.3	0.12
Cholesterol (mmol/L)	4.7	4.3–5.1	5.1	4.9–5.4	0.1
Triglycerides (mmol/L)	1.1	0.9–1.2	1.2	0.9–1.5	0.39
LDL (mmol/L)	2.7	2.3–3.0	3.1	2.9–3.4	0.09
HDL (mmol/L)	1.5	1.4–1.7	1.5	1.4–1.6	0.88
CRP (mg/L)	1.8	1.1–2.5	2.2	1.5–2.8	0.47
Stool					
FC (µg/g) (Median)	14.8	(IQR) 6.3–30.2	17.4	(IQR) 8.5–74	0.25

Legend: Data are mean and CI (95%). BMI: Body Mass Index, CI: confidence interval, CRP: c-reactive protein, FC: faecal calprotectin, HbA1c: glycated hemoglobin, HC: Healthy Control, HDL: high-density lipoprotein, IQR: interquartile range, LDL: low-density lipoprotein. * = statistically significant finding, *p* < 0.05.

**Table 2:** Average physical activity and daily dietary intake in the HC and IBD participant groups.

Parameter	HC	SD±	IBD	SD±	*p*-Value	CI
Steps (avg/day)	10,942	2746	9135	3784	0.047 *	25–33,588
Estimated BMR kJ/day	6816	1088	6916	1381	0.75	−532.2–731.9
Recorded energy intake (kJ/day)	9769	2652	9184	2342	0.33	−1774–602.6
Average PAL (EI/BMR)	1.42	0.2	1.35	0.3	0.12	−0.22–0.08
Under-reporting (EI/BMR < 1.07)/ did not record	4		15		0.35	n/a (chi-square test)
Protein (g)	100	26	95	29	0.5	−18–9
Fat (g)	92	25	89	27	0.59	−16–9
Saturated Fat (g)	35	10	33	12	0.43	−8–3
Trans Fat (g)	1.4	0.5	1.3	0.5	0.69	−0.3–0.2
PUFA (g)	12	3.9	12	4.7	0.66	−2–3
MUFA (g)	33	9.4	33	12.0	0.97	−7–5
Carbohydrate (g)	222	82	220	60	0.93	−34–31
Sugar (g)	97	44	87	33	0.33	−30–10
Alcohol (g)	16	14	12	16	0.28	−11–3
Dietary fibre (g)	25	10	23	10	0.43	−7–292
Sodium (mg)	2463	690	3070	3342	0.38	−769–1983
Calcium (mg)	919	295	815	330	0.19	−259–53
Zinc (mg)	10	3	10	3	0.48	−2–1
Folate (µg)	594	318	575	245	0.77	−150–112
Wholegrains (serves)	2.08	1.7	1.0	0.9	<0.01 *	−1.7–−0.5
Fruit (serves)	1.1	1.2	1.0	0.8	0.73	−0.5–0.4
Vegetables (serves)	4.4	2.7	4.7	3.8	0.72	−1.4–2
Caffeine (mg)	267	248	215	253	0.4	−175–70
Red meat (serves)	0.5	0.4	0.6	0.7	0.48	−0.2–0.4
Poultry (serves)	0.5	0.4	0.5	0.5	0.95	−0.2–0.2
Egg (serves)	0.3	0.2	0.5	1.4	0.43	−0.3–0.8
Processed meat (serves)	0.2	0.2	0.2	0.3	0.65	−0.1–0.2
Seafood (serves)	0.3	0.2	0.3	0.3	0.73	−0.1–0.2
Added sugars (tsp)	8	7.9	7	5.1	0.69	−4–2
Discretionary food (serves)	4.8	3.3	4.8	2.5	0.95	−1.3–1.4
HEIFA-2013 (score)	53	11	49	10	0.21	−8–2

Legend: BMR: basal metabolic rate, CI: confidence index, EI: energy intake, HC: Healthy Controls, HEIFA-13: Healthy eating index for Australian Adults, n/a: not applicable, MUFA: monounsaturated fatty acids, PAL: physical activity level, PUFA: polyunsaturated fatty acid. * = statistically significant finding, *p* < 0.05.

**Table 3:** In the IBD group: mean dietary intake, serum metabolic parameters, anthropometric parameters and stool microbial alpha diversity indices of participants with clinical disease flare-up at 18.5 months (median) follow-up compared with those who remained in clinical remission.

Parameter	Clinical Disease Flare-Up (*n* = 13)	Clinical Remission (*n* = 43)	*p*	CI
Dietary intake (average per day)				
Discretionary foods (serves)	4.5	4.9	0.62	−1.2–2.01
Added sugar (g)	6.8	7.6	0.64	−2.5–4.1
Total fat (g)	83.7	88.8	0.54	−11.5–21.6
Saturated fat (g)	31.0	32.6	0.66	−5.9–9.3
Trans fat (g)	1.2	1.4	0.67	−0.1–0.5
Processed meat (serves)	1.7	2.0	0.88	−0.2–0.2
Fruit (serves)	1.4	0.9	0.50	−1.0–0
Vegetables (serves)	4.5	4.8	0.89	−2.3–2.8
Fiber (g)	23.2	23.5	0.95	−6.1–6.6
HEIFA-2013 (score)	53.4	46.6	0.50	−15.2–1.4
Serum metabolic profile				
Insulin (IU/L)	6.7	6.1	0.64	−3.1–1.9
HbA1c (%)	4.9	4.9	0.49	−0.2–0.3
Cholesterol (mmol/L)	4.7	5.1	0.11	0–1.3
Triglycerides (mmol/L)	1.1	1.2	0.73	−0.6–0.8
HDL-cholesterol (mmol/L)	1.5	1.5	0.71	−0.2–0.3
LDL-cholesterol (mmol/L)	2.6	3.1	0.54	0–1.14
Anthropometric parameters				
BMI (kg/m^2^)	23.9	25.6	0.22	−1.0–4.4
Waist Circumference (cm)	80.7	85.5	0.35	−5.4–14.8
Waist-to-Hip Ratio	0.8	0.9	0.29	0–0
Stool microbial diversity indices				
Species Richness	23.4	20.7	0.30	−7.9–2.5
Species evenness	0.6	0.6	0.80	−0.1–0
Shannon’s diversity index	3.1	3.1	0.57	−0.5–0.3

Legend: Data are mean values, with *t*-test used to examine for differences between groups. BMI: Body Mass Index, CI: Confidence interval, HbA1c: glycated haemoglobin, HDL: high-density lipoprotein, HEIFA-13: Healthy eating index for Australian Adults, LDL: low-density lipoprotein, *p*: *p*-value, species richness: higher number greater unique species within the sample, species evenness: number 0–1, approaching 1 = species have same abundance, approaching 0 = species vary, Shannon’s diversity index: a measure of diversity which takes into account abundance and evenness of a species.

**Table 4:** Correlations between dietary intake and anthropometry and glucose control in the IBD and HC groups corrected for age, gender and total caloric intake and *p*-values adjusted for false discovery rate.

	BMI						WC						WHR					
	IBD			HC			IBD			HC			IBD			HC		
	r	*df*	*p*-adj	r	*df*	*p*-adj	r	*df*	*p*-adj	r	*df*	*p*-adj	r	*df*	*p*-adj	r	*df*	*p*-adj
Discretionary Foods	0.33	50	0.21	0.21	19	0.83	0.3	43	0.09	0.21	18	0.37	0.32	43	0.1	0.37	18	0.87
Added Sugar	0.32	50	0.23	−0.15	19	0.9	0.31	43	0.09	−0.28	18	0.24	0.31	43	0.43	−0.28	18	0.87
Saturated Fat	0.37	50	0.04 *	0.02	19	0.92	0.39	43	0.04 *	0.34	18	0.15	0.24	43	0.22	0.06	18	0.96
Trans Fat	0.19	50	0.23	0.26	19	0.78	0.2	43	0.26	0.25	18	0.29	0.27	43	0.18	−0.01	18	0.96
Processed Meat	0.3	50	0.1	0.59	19	0.05 *	0.25	43	0.15	0.58	18	<0.01 *	0.16	43	0.39	0.25	18	0.87
Fruit	−0.24	50	0.15	−0.29	19	0.78	−0.18	43	0.26	−0.31	18	0.19	−0.22	43	0.23	−0.1	18	0.96
Vegetable	−0.25	50	0.14	−0.09	19	0.9	−0.27	43	0.73	−0.31	18	0.19	−0.28	43	0.61	−0.17	18	0.96
Fibre	−0.45	50	0.01 *	−0.06	19	0.9	−0.44	43	0.02 *	0.13	18	0.59	−0.45	43	0.02 *	0.03	18	0.96
HEIFA-13	−0.27	50	0.13	−0.11	19	0.9	−0.25	43	0.15	−0.23	18	0.32	−0.32	43	0.1	−0.12	18	0.96
	**Fasting Glucose**						**HbA1c**						**Insulin**					
	**IBD**			**HC**			**IBD**			**HC**			**IBD**			**HC**		
	**r**	***df***	***p*-adj**	**r**	***df***	***p*-adj**	**r**	***df***	***p*-adj**	**r**	***df***	***p*-adj**	**r**	***df***	***p*-adj**	**r**	***df***	***p*-adj**
Discretionary Foods	0.13	49	0.55	0.27	19	0.54	0	50	0.97	−0.03	18	0.97	0.27	47	0.13	0.12	19	0.93
Added Sugar	0.03	49	0.93	0.05	19	0.84	0.01	50	0.97	−0.31	18	0.97	0.32	47	0.09	−0.03	19	0.93
Saturated Fat	0.01	49	0.94	0.24	19	0.54	0.05	50	0.9	0.12	18	0.97	0.28	47	0.13	0.02	19	0.93
Trans Fat	0.14	49	0.55	0.17	19	0.71	0.26	50	0.14	−0.03	18	0.97	0.25	47	0.14	0.1	19	0.93
Processed Meat	0.15	49	0.55	−0.05	19	0.84	0.26	50	0.14	−0.26	18	0.97	0.22	47	0.17	0.53	19	0.72
Fruit	−0.28	49	0.14	−0.3	19	0.54	−0.31	50	0.11	0.04	18	0.97	0.03	47	0.83	−0.32	19	0.72
Vegetable	−0.11	49	0.55	0.06	19	0.84	−0.19	50	0.32	−0.01	18	0.97	−0.26	47	0.13	0.1	19	0.93
Fibre	−0.39	49	0.05 *	−0.24	19	0.54	−0.34	50	0.11	0.26	18	0.97	−0.36	47	0.09	−0.06	19	0.93
HEIFA-13	−0.32	49	0.09	−0.25	19	0.54	−0.15	50	0.44	−0.17	18	0.97	−0.16	47	0.29	−0.21	19	0.93

Legend: *: statistically significant, BMI: Body Mass Index, *df*: degrees of freedom, HbA1c: glycated haemoglobin, HC: Healthy Control, HEIFA-13: Healthy eating index for Australian Adults, *p*-adj: *p*-value adjusted for false discovery rate using the Benjamini–Hochberg procedure, r: partial correlation coefficient adjusted for variables of age, gender and average energy input, WC: waist circumference, WHR: waist-to-hip ratio.

**Table 5:** Correlations between anthropometric parameters, measures of glucose control and lipid profile in the IBD group corrected for the variables of age and gender.

	BMI						WC						WHR					
	IBD			HC			IBD			HC			IBD			HC		
	r	*df*	*p*-adj	r	*df*	*p*-adj	r	*df*	*p*-adj	r	*df*	*p*-adj	r	*df*	*p*-adj	r	*df*	*p*-adj
Fasting glucose	0.17	52	0.32	0.47	19	0.12	0.25	46	0.13	0.43	18	0.12	0.28	46	0.09	0.15	18	0.69
HbA1c	0.21	53	0.21	−0.22	18	0.42	0.25	46	0.13	0.34	17	0.21	0.27	46	0.09	0.38	17	0.69
Insulin	0.48	50	<0.01 *	0.44	18	0.12	0.55	45	<0.01 *	0.42	18	0.123	0.55	45	<0.01 *	−0.1	18	0.69
LDL	0.11	52	0.5	0.45	18	0.12	0.14	45	0.34	0.58	18	0.03 *	0.24	45	0.13	0.15	18	0.69
HDL	−0.44	52	<0.01 *	−0.03	18	0.91	−0.3	46	0.09	0.2	18	0.4	−0.04	46	0.77	0.02	18	0.69
Cholesterol	0	52	0.98	0.39	19	0.14	0.14	46	0.34	0.58	18	0.03 *	0.32	46	0.07	0.24	18	0.69
Triglycerides	0.26	53	0.12	0.21	19	0.42	0.36	46	0.04 *	0.23	18	0.37	0.57	46	<0.01 *	−0.13	18	0.69

Legend: *: statistically significant, BMI: Body Mass Index, *df*: degrees of freedom, HbA1c: glycated haemoglobin, HDL: high-density lipoprotein, LDL: low-density lipoprotein, *p*-adj: *p*-value adjusted by the Benjamini–Hochberg procedure to control for false discovery rate, r: partial correlation coefficient adjusted for variables of age, gender and average energy input, WC: waist circumference, WHR: waist-to-hip ratio.

**Table 6:** Correlations between serum metabolic profile and anthropometric parameters with c reactive protein and faecal calprotectin in the IBD and HC groups corrected for age and gender.

	CRP						FC					
	IBD			HC			IBD			HC		
	r	*df*	*p*-adj	r	*df*	*p*-adj	r	*df*	*p*-adj	r	*df*	*p*-adj
	Serum metabolic profile											
Fasting Glucose	0.15	52	0.29	0.57	20	0.07	0.3	39	0.3	−0.15	6	0.8
Insulin	0.35	50	0.03 *	−0.13	20	0.7	0.15	39	0.4	−0.4	6	0.57
HbA1c	0.17	53	0.25	0.11	19	0.7	0.2	40	0.3	0.2	6	0.8
Cholesterol	0.17	53	0.25	0.25	20	0.5	−0.24	40	0.3	−0.69	6	0.57
Triglycerides	0.3	53	0.04 *	0.32	20	0.5	0.22	40	0.3	−0.77	6	0.57
HDL	−0.42	53	0.01 *	0.08	20	0.72	−0.27	40	0.3	0.05	6	0.91
LDL	0.23	52	0.11	0.25	20	0.5	−0.26	39	0.3	−0.59	6	0.57
	Anthropometric measures											
BMI	0.4	53	0.02 *	0.28	20	0.5	0.21	40	0.3	−0.31	6	0.64
WC	0.4	46	0.02 *	0.3	19	0.5	0.15	36	0.4	−0.39	6	0.57
WHR	0.29	46	0.09	0.11	19	0.7	0.06	36	0.72	−0.42	6	0.57
	Inflammatory markers											
FC	0.27	40	0.12	−0.4	6	0.5						

Legend: *: statistically significant, adj-*p*: *p*-value adjusted for false discovery rate using the Benjamini–Hochberg procedure, BMI: Body Mass Index, *df*: degrees of freedom, CRP: c-reactive protein, FC: faecal calprotectin, HbA1c: glycated haemoglobin, HC: healthy control, HDL: high-density lipoprotein, LDL: low-density lipoprotein, r: partial correlation coefficient adjusted for variables of age and gender.

**Table 7:** Correlation between dietary intake, serum metabolic profile, anthropometric parameters and inflammatory markers with and measures of stool microbial alpha diversity metrics in the IBD and HC groups, corrected for age and gender and *p*-values adjusted for false discovery rate.

	Species Richness						Species Evenness						Shannon’s Diversity					
	IBD			HC			IBD			HC			IBD			HC		
	r	*df*	*p*-adj	r	*df*	*p*-adj	r	*df*	*p*-adj	r	*df*	*p*-adj	r	*df*	*p*-adj	r	*df*	*p*-adj
	Dietary intake																	
Discretionary Foods	−0.33	47	0.10	0.2	19	0.91	−0.17	47	0.30	−0.14	19	0.96	−0.27	47	0.65	−0.05	19	0.99
Added Sugar	−0.4	47	0.10	0.04	19	0.91	−0.31	47	0.11	−0.7	19	0.96	−0.39	47	0.03 *	−0.04	19	0.99
Processed Meat	−0.37	47	0.10	−0.04	19	0.91	−0.39	47	0.04 *	−0.09	19	0.96	−0.43	47	0.02 *	−0.04	19	0.99
Saturated Fat	−0.15	47	0.40	0.54	19	0.21	−0.26	47	0.15	0.1	19	0.96	−0.22	47	0.18	0.19	19	0.99
Trans Fat	−0.13	47	0.44	0.39	19	0.84	−0.08	47	0.62	0.08	19	0.96	−0.09	47	0.64	0.15	19	0.99
Fruit	0.19	47	0.30	−0.15	19	0.91	−0.03	47	0.84	0.2	19	0.96	0.04	47	0.79	0.09	19	0.99
Vegetables	0.18	47	0.30	−0.04	19	0.91	0.29	47	0.13	0.08	19	0.96	0.27	47	0.67	0.09	19	0.99
Fibre	0.31	47	0.10	−0.13	19	0.91	0.26	47	0.15	0.06	19	0.96	0.3	47	0.12	0.04	19	0.99
HEIFA-13	0.18	47	0.30	−0.09	19	0.91	0.14	47	0.39	0.01	19	0.96	0.18	47	0.26	0.04	19	0.99
	Serum metabolic profile																	
Fasting plasma glucose	−0.24	47	0.21	0.14	19	0.91	−0.22	47	0.21	−0.02	19	0.96	−0.27	47	0.16	0.07	19	0.99
Insulin	−0.33	46	0.30	−0.13	19	0.91	−0.45	46	0.02 *	0.03	19	0.96	−0.45	47	0.02	0.01	19	0.99
HbA1c	−0.3	47	0.10	0.24	18	0.91	−0.18	47	0.30	0.21	18	0.96	−0.24	47	0.18	0.21	19	0.99
Cholesterol	−0.21	47	0.26	−0.05	19	0.91	−0.15	47	0.36	−0.01	19	0.96	−0.2	47	0.24	−0.01	19	0.99
Triglycerides	−0.24	47	0.21	−0.3	19	0.91	−0.32	47	0.11	0.05	19	0.96	−0.32	47	0.11	0.10	19	0.99
HDL-cholesterol	0.11	47	0.50	0.29	19	0.91	0.28	47	0.13	0.14	19	0.96	0.23	47	0.18	0.14	19	0.99
LDL-cholesterol	−0.23	47	0.23	−0.12	19	0.91	−0.22	47	0.21	−0.07	19	0.96	−0.24	47	0.18	−0.08	19	0.99
	Anthropometric measures																	
BMI	−0.31	47	0.01	−0.03	19	0.91	−0.34	47	0.09	0.02	20	0.96	−0.36	47	0.04 *	0.07	19	0.99
Waist Circumference	−0.28	42	0.61	0.17	18	0.91	−0.2	42	0.29	0.21	18	0.96	−0.25	47	0.18	0.26	18	0.99
Waist-to-Hip Ratio	−0.34	42	0.01	0.17	18	0.91	−0.14	42	0.40	−0.02	18	0.96	−0.25	47	0.18	0.05	18	0.99
	Inflammatory markers																	
CRP	−0.24	47	0.21	0.16	19	0.91	−0.43	47	0.02 *	−0.14	19	0.96	−0.41	47	0.03 *	−0.02	19	0.99
Fecal calprotectin	−0.12	37	0.50	−0.07	5	0.91	−0.29	37	0.15	0.75	5	0.96	−0.22	37	0.24	0.77	5	0.99

Legend: *: statistically significant, adj-*p*: *p*-value adjusted for false discovery rate using the Benjamini–Hochberg procedure, BMI: Body Mass Index, CRP: c-reactive protein, *df*: degrees of freedom FC: faecal calprotectin, HbA1c: glycated haemoglobin, HC: healthy control, HEIFA-13: Healthy eating index for Australian Adults, HDL: high-density lipoprotein, LDL: low-density lipoprotein, r: partial correlation coefficient adjusted for variables of age and gender, species richness: higher number greater unique species within the sample, species evenness: number 0–1, approaching 1 = species have same abundance, approaching 0 = species vary, Shannon’s diversity index: a measure of diversity which takes into account abundance and evenness of a species.

## Data Availability

The original contributions presented in the study are included in the article and Supplementary Materials, further inquiries can be directed to the corresponding author.

## Supplementary Materials

The following supporting information can be downloaded at https://www.mdpi.com/article/10.3390/nu16193233/s1, Table S1: IBD participant group clinical characteristics; Table S2: (A) PERMANOVA analysis of variables that associated with stool microbial beta diversity in thewhole group, (IBD and HC groups combined, *df* = 1, 65), (B) PERMANOVA analysis of variables that associated with stool microbial beta diversity in theIBD group (*df* = 1, 43); Figure S1: Bar graphs depict stool microbiome alpha diversity components: species richness (A), species evenness (B) and Shannon’s diversity index (C) comparing the IBD and HC cohorts. Principal Coordinates Analysis (PCA) plot (D) comparing the stool microbiome bacteria beta diversity of the IBD v HC cohort.
