# Supplementary material for: Dietary Determinants of Metabolic and Gut Microbial Health in Patients with Inflammatory Bowel Disease
Source: Nutrients. 2024 Sep 24;16(19):3233. doi: 10.3390/nu16193233 (PMC11479224; doi:10.3390/nu16193233)
Supplement: Supplementary file 1 [file nutrients-16-03233-s001.zip › nutrients-3127792-supplementary.pdf]

Supplementary Materials

Supplementary Table S1. IBD participant group clinical characteristics

		n= 57	Percent
**Crohn's disease**	**Total**	31	54.4%
	**L2 (Colonic)**	15	
	**L3 (Ileocolonic)**	16	
	**plus L4 (upper GI)**	2	
	**B1 (non stricturing, non-penetrating)**	28	
	**B2 (stricturing)**	0	
	**B3 (penetrating)**	3	
	**Perianal disease**	14	
	**CDAI**	27.8 (± 23.06)	
**Ulcerative cholitis**	**Total**	26	45.6%
	**E2**	13	
	**E3**	13	
	**Clinical MAYO**	0.15 (± 0.46)	
	**MAYO= 0**	23	
	**MAYO= 1**	2	
	**MAYO= 2**	1	
**Faecal calprotectin (µg/g) (median)**		16.3 (Range 5-800)	
**Biologic use**		45	79.0%
	**1**	31	
	**2**	10	
	**3**	4	
**Previous hospitalisation**		39	68.4%
**Number of previous steroid courses (Median)**		3 (0-15)	
**Previous resection**		2	3.5%
**Current treatment**	**Biologics**	45	79.0%
	**Immunomodulator**	25	43.8%
	**Combination therapy (Biologic + Immunomodulator)**	21	36.8%
	**5-ASA**	17	29.8%
**Duration of follow up median (months)**		18.5 (12-24)	
**Flare within 12 months**		7	12.5%

**Flare at any point during follow up**		13	23.2%
**Mean months to flare (in those that flared)**		13	

Legend: 5-ASA:5-aminosalicylic acid, CDAI: Crohn's Disease Activity Index, Classification of Ulcerative Colitis, E1: Proctitis, E2: Left-sided, E3 pancolitis, Clinical Mayo Score: disease activity index UC, assesses rectal bleeding, stool frequency and physician global assessment. min score: 0 = no symptoms, max score: 9= severe disease. Montreal classification of Crohn's disease: L2: Colonic, L3: Ileocolonic, L4: Upper Gastrointestinal Crohn's in addition to colonic disease, B1: Non-Stricturing, Non-Penetrating, B2: Stricturing, B3: Penetrating, SD: standard deviation.

Supplementary Table S2.

A) PERMANOVA analysis of variables that associated with stool microbial beta diversity in the whole group, (IBD and HC groups combined, *df* = 1, 65)
B) PERMANOVA analysis of variables that associated with stool microbial beta diversity in the IBD group (*df* = 1, 43)

A)

PERMANOVA: whole cohort			
Variable	Pseudo-F	P(perm)	Perms
Age	1.37	0.06	9866
BMI	1.19	0.18	9850
Insulin	1.66	0.01*	9866
Processed meat	1.42	0.06	9871
Study arm	3.23	<0.01*	9876
Sex	1.03	0.39	9862

B)

PERMANOVA: IBD group			
Variable	Pseudo-F	P(perm)	Perms
Age	1.29	0.11	9873
BMI	1.28	0.12	9880
Insulin	1.42	0.07	9885
Processed meat	1.38	0.08	9866
Sex	0.90	0.62	9855

Legend: *: statistically significant, *df*: Degrees of freedom, p(perm): Probability result generated using a PERMANOVA multivariate analysis of variance calculation, HEIFA-13: Healthy eating index for Australian Adults, PERMANOVA: Permutational multivariate analysis of variance, Perms: a calculation performed using a PERMANOVA multivariate analysis, calculation, Pseudo-F: ratio of

the between-cluster variation to the within-cluster variation. The larger the pseudo-F value the greater the difference between samples compared.

Supplementary Figure S1.
Bar graphs depict stool microbiome alpha diversity components: species richness (A), species evenness (B) and Shannon's diversity index (C) comparing the IBD and HC cohorts. Principal Coordinates Analysis (PCA) plot (D) comparing the stool microbiome bacteria beta diversity of the IBD v HC cohort,
The x-axis represents the PCO that explains the largest data change, and the PCO on the y-axis accounts for the second largest proportion of the data change. The spatial separation of sample points represents the distance between samples, dots closer to one another are more similar than those further apart.

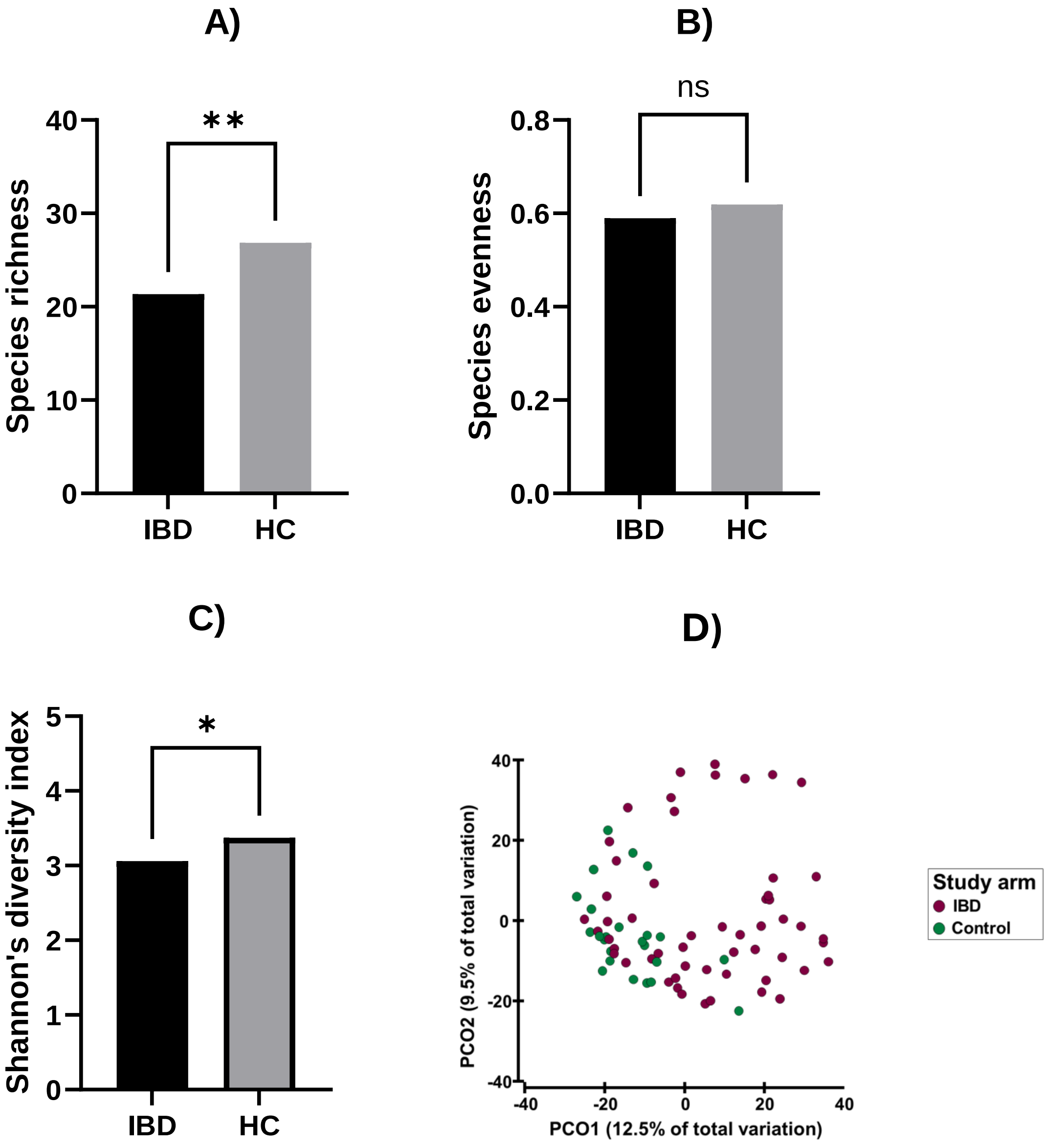

Legend :

A) OTU: operational taxonomic units, S1: Stool sample, *: p 0.029, **: $p < 0.01$, ns: not significant.

B) PCO: Principal Coordinates Analysis
